# Sleep Quality Detection Based on EEG Signals Using Transfer Support Vector Machine Algorithm

**DOI:** 10.3389/fnins.2021.670745

**Published:** 2021-04-23

**Authors:** Wu Wen

**Affiliations:** Chongqing Technology and Business Institute, Chongqing, China

**Keywords:** sleep quality detection, EEG signal, discrete wavelet transform, transfer support vector machine, national sleep research resource library

## Abstract

**Background:**

In recent years, with the acceleration of life rhythm and increased pressure, the problem of sleep disorders has become more and more serious. It affects people’s quality of life and reduces work efficiency, so the monitoring and evaluation of sleep quality is of great significance. Sleep staging has an important reference value in sleep quality assessment. This article starts with the study of sleep staging to detect and analyze sleep quality. For the purpose of sleep quality detection, this article proposes a sleep quality detection method based on electroencephalography (EEG) signals.

**Materials and Methods:**

This method first preprocesses the EEG signals and then uses the discrete wavelet transform (DWT) for feature extraction. Finally, the transfer support vector machine (TSVM) algorithm is used to classify the feature data.

**Results:**

The proposed algorithm was tested using 60 pieces of data from the National Sleep Research Resource Library of the United States, and sleep quality was evaluated using three indicators: sensitivity, specificity, and accuracy. Experimental results show that the classification performance of the TSVM classifier is significantly higher than those of other comparison algorithms. This further validated the effectiveness of the proposed sleep quality detection method.

## Introduction

As an important physiological phenomenon and a necessary physiological process, sleep is considered to be a resting state with a greatly reduced response capacity (Siegel, 2005). The body eliminates fatigue through sleep, restores mental and physical strength, and maintains a good state. At present, the pace of social life is fast, and sleep disorders have become an increasingly common problem. The problem of sleep disturbance will have a negative impact on the body’s alertness and attention, causing patients to have adverse consequences due to reduced alertness. Many physiological functions change during sleep, such as decreased skeletal muscle tension, slower breathing, and decreased visual, auditory, tactile, and other sensory sensitivities. These changes also vary in different sleep stages. Changes in physiological functions during sleep lead to corresponding changes in electrophysiological signals, and sleep research has also been carried out.

Sleep quality assessment is an important branch of sleep research and an integral part of sleep neurobiology research. Because sleep neurobiology is closely related to cognitive neuroscience, sleep quality assessment also helps in studying various neurocognitive functions such as learning and memory. This shows that sleep quality assessment plays an important role in sleep research. Surveys indicate that most people suffer from poor sleep quality due to physical or psychological problems. And poor sleep quality will produce further positive feedback on the original physical and psychological problems, which will continue to deteriorate. At present, the burden on doctors for the diagnosis and detection of such diseases is relatively heavy. Especially for the acquisition and processing of patients’ night sleep data, the help of automation and digital technology is very much needed. Therefore, the research in this paper will have irreplaceable clinical value and practical significance. At present, the research direction of sleep quality mainly focuses on the study of sleep staging. With the continuous development of computer technology, many machine learning methods had been proposed and used in the medical applications (Bezdek, 1980, 1981; Hall et al., 1992; Ahmed et al., 2002; Pedrycz, 2002; Chen and Zhang, 2004; Chuang et al., 2006; Weijer and Gevers, 2006; Jing et al., 2007; Cleuziou et al., 2009; Gu and Zhou, 2009; Zhu et al., 2009; Krinidis and Chatzis, 2010; Hall and Goldgof, 2011; Ji et al., 2011; Jiang et al., 2012, 2014, 2020; Yu et al., 2012; Chen et al., 2013; Gong et al., 2013; Thanh and Wu, 2013; Li et al., 2014; Elazab et al., 2015; Okita et al., 2015; Qian et al., 2015; Wang et al., 2015, 2019; Zheng et al., 2015; Devi and Setty, 2018; Lee et al., 2018; Rosati et al., 2018; Cai et al., 2019; Gu et al., 2019; Chrobak et al., 2020; Kumar et al., 2020; Liu et al., 2020; Singh et al., 2020; Sunjana and Azizah, 2020; Yin et al., 2020; Zhang et al., 2020). The sleep staging method has evolved from the traditional visual observation method to the automatic staging method based on extracting physiological signal features. The accuracy and efficiency of sleep staging are greatly improved. The process of automatic sleep staging algorithm mainly includes signal preprocessing, feature extraction, sleep stage classification, and result output. The commonly used models for sleep stage classification are machine learning algorithms such as support vector machine (SVM) (Doroshenkov et al., 2007; Hsu and Yang, 2013; Qian et al., 2016a,b, 2017, 2018a,b, 2020; Jiang et al., 2017a,b, 2019; Xia et al., 2019). The methods commonly used to extract the characteristic parameters of sleep staging from electroencephalography (EEG) data mainly include wavelet transform and other methods (Alessandro et al., 2001; Kannathal et al., 2005; Srinivasan et al., 2005; Mohseni et al., 2006; Subasi, 2007; Bruzzo et al., 2008; Albayrak and Koklukaya, 2009; Yuen et al., 2009; Fathima et al., 2010; Geng et al., 2011; Gandhi et al., 2012; Sen and Peker, 2013). Table 1 shows the progress of sleep staging research in recent years.

**TABLE 1:** Research progress in sleep staging.

References	Type of data	Characteristic parameters	Classification model
Huang et al. (2014)	EEG	STFT	RVM
Fraiwan et al. (2010)	EEG	Multiwavelet time–frequency entropy	LDA
Fraiwan et al. (2012)	EEG	Time–frequency analysis	RF
Baja and Pachori (2013)	EEG	Pseudo Wigner–Ville distribution	LS-SVM
Long et al. (2014)	EEG	Viewable	SVM
Koch and Christensen (2014)	Respiratory signal	Amplitude, depth characteristics	LDA
Kayikcioglu et al. (2015)	EEG, EOG	Dirichlet distribution	SVM
Hassan and Bhuiyan (2016)	EEG	AR coefficient	PLS
Hassan and Bhuiyan (2016)	EEG	EMD	AdaBoost
Lajnef et al. (2015)	EMG, EOG	Energy characteristics	SVM

**EEG, electroencephalography; EOG, electrooculography; EMG, electromyography; STFT, short-time Fourier transform; AR, autoregressive; EMD, empirical mode decomposition; RVM, relevance vector machine; LDA, linear discriminant analysis; RF, radiofrequency; LS-SVM, least squares support vector machine; PLS, partial least squares.**

In this study, EEG signals were selected. After preprocessing and feature extraction, the signals were classified using the transfer support vector machine (TSVM) classifier. The performance of the algorithm is evaluated from the three indicators of sensitivity, specificity, and accuracy. The work of this paper is summarized as follows:

(1) The transfer mechanism is introduced into the classic SVM algorithm to obtain the transfer learning SVM (TL-SVM) classification model. Since the model can use the source domain dataset to guide the classification of the target domain dataset, the accuracy of the classification is improved to a certain extent.
(2) A sleep EEG recognition method based on TL-SVM is proposed. Experiments show that this method is feasible and effective for sleep quality detection.

## Background

### Introduction to Sleep Staging

This article selects EEG signals for the study of sleep quality; therefore, here, we focus on analyzing the role of EEG in sleep staging. We mainly describe sleep staging and the relationship between EEG and sleep.

**FIGURE 1:**
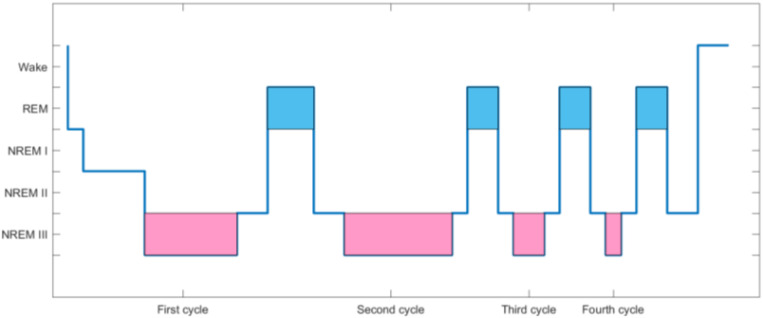
Adult sleep structure diagram.

According to the American Academy of Sleep Medicine (AASM) staging criteria, a normal sleep cycle can be divided into two stages, namely, the non-rapid eye movement (NREM) period and the rapid eye movement (REM) period. According to the depth of sleep, NREM is further divided into stages I–III. The above-mentioned stages recur periodically during sleep of normal people all night. When going to sleep, a normal person’s sleep stage first enters the NREM stage, gradually transitioning from stage I to III, and then enters the REM stage at stage II or III of NREM. The sleep stage enters the REM stage from the NREM stage, representing a complete sleep cycle. Normal adults have about four to six sleep cycles all night. Under normal circumstances, the sleep time of stage I in normal adults accounts for about 5% of a whole night’s sleep, stage III in NRE accounts for about 50%, stage II in NRE accounts for 20%, and the REM stage accounts for 25%. Figure 1 is a normal adult sleep structure diagram.

The red area in Figure 1 represents the NREMIII stage, which is the stage of deep sleep. The yellow area is the REM period where “dreams” often appear. As the black line drops, it means deeper sleep. The higher the position of the black line, the lighter the sleep. During the sleep cycle, EEG will change correspondingly with the change of sleep stage.

### Sleep EEG Signal

During sleep, the brain often has rhythm, amplitude, frequency, and other EEG rhythms. The thalamus is the generating part of the EEG rhythm. Its main function is to receive excitement from the brain stem to form a thalamus cortical circuit, thereby regulating the level of neuron excitement. Thalamic neurons have low-threshold calcium channels. During human sleep, thalamic afferent stimulation is low and membrane potential is low, causing calcium channel opening, a large amount of calcium ion influx, forming a short excitatory postsynaptic potential (EPSP). Because a large number of inhibitory neurons in the thalamus block the afferent stimulation, a series of longer inhibitory postsynaptic potentials (IPSP) will follow the EPSP to form a group of EPSP–IPSP. This goes on repeatedly to form the EEG rhythm. The EEG rhythm with typical characteristics during sleep can be used as a basis for judging the sleep stage and diagnosing sleep diseases. Taking adults as an example, Table 2 shows five typical rhythms. It can be seen from these five types of rhythms that different EEG rhythms have large differences in frequency, amplitude, shape, etc., which is a good electrophysiological basis for sleep staging.

**TABLE 2:** Introduction to five types of rhythm.

Slow wave Θ	During the transition period from just falling asleep to light sleep, slow-wave Θ activity repeatedly bursts. And, often adjacent to the top wave, there is no specific shape standard. The average frequency is generally 5–7 Hz, and it appears more in the center of the head and in the top area.
Top wave	The top wave is a sign of the N1 phase, which can be extended to the N2 phase. The maximum amplitude often appears in the cranial region, and the frequency is generally 3–8 Hz. A typical top wave is symmetrically synchronized on both sides and has a sharp shape.
σ rhythm	The σ rhythm is a sign of the N2 period and can be continued to the N3 period, which lasts more than 0.5 s. Its maximum amplitude often appears in the cranial area and can reach the frontal area, central area, and apical area on both sides of the head. The σ rhythm is generally a 12- to 14-Hz spindle-shaped wave, so it is also called a spindle wave.
κ synthetic wave	κ synthetic waves often appear in the N2 phase and can continue to the N3 phase, mainly distributed in the apical or frontal area of the head. The waveform is similar to the top wave, but is wider. It is a steep negative wave followed by a positive wave, often followed by a series of 12- to 14-Hz σ rhythms. Synthetic waves are often induced by external stimuli such as sound and touch.
α rhythm	The α rhythm is a symbolic rhythm of awake state. It often appears in the back of the head and can spread to the central region, the middle temporal region, or the troubled roof. The α rhythm is generally 9–11 Hz.

## Sleep Quality Detection Based on EEG Signals

### Sleep Quality Testing Process

**FIGURE 2:**
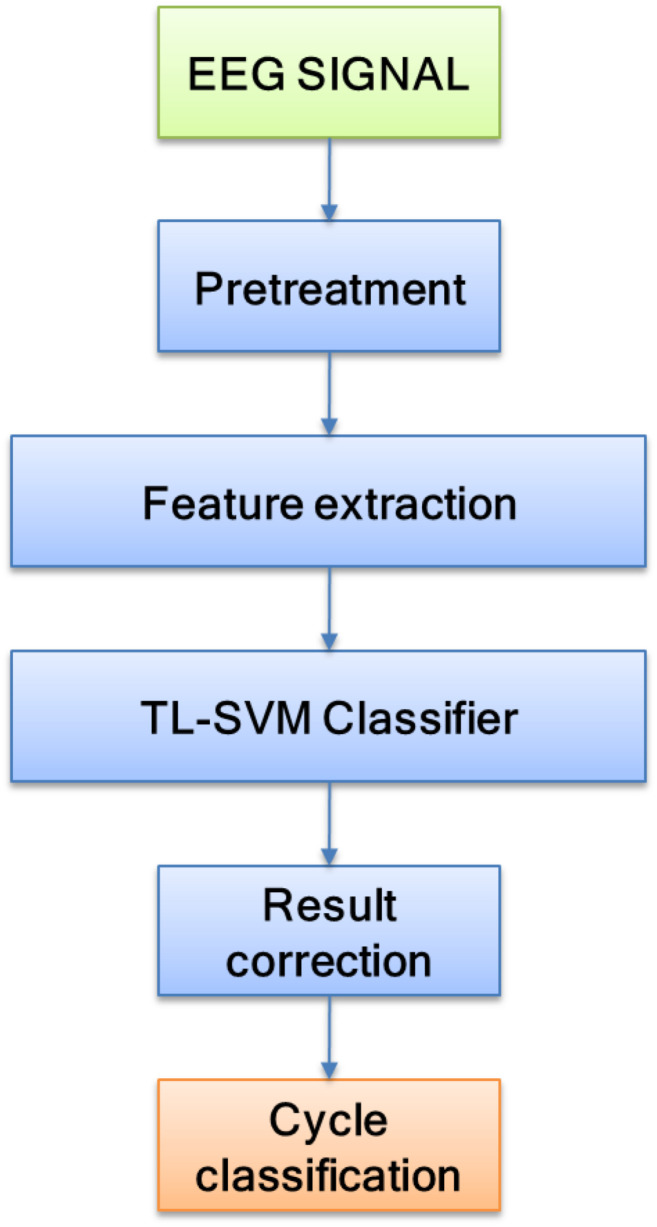
Flowchart of the sleep staging method.

The core of sleep quality detection lies in sleep staging. Being able to design a highly accurate sleep staging algorithm can effectively promote the inspection of sleep quality. The flow of the sleep staging method is shown in Figure 2. In this study, EEG signals were selected as the input signal source, and preprocessing and feature extraction were performed on the EEG signals. Finally, the TSVM classifier and preliminary staging results were corrected to complete the sleep staging.

### Brainwave Pretreatment

**FIGURE 3:**
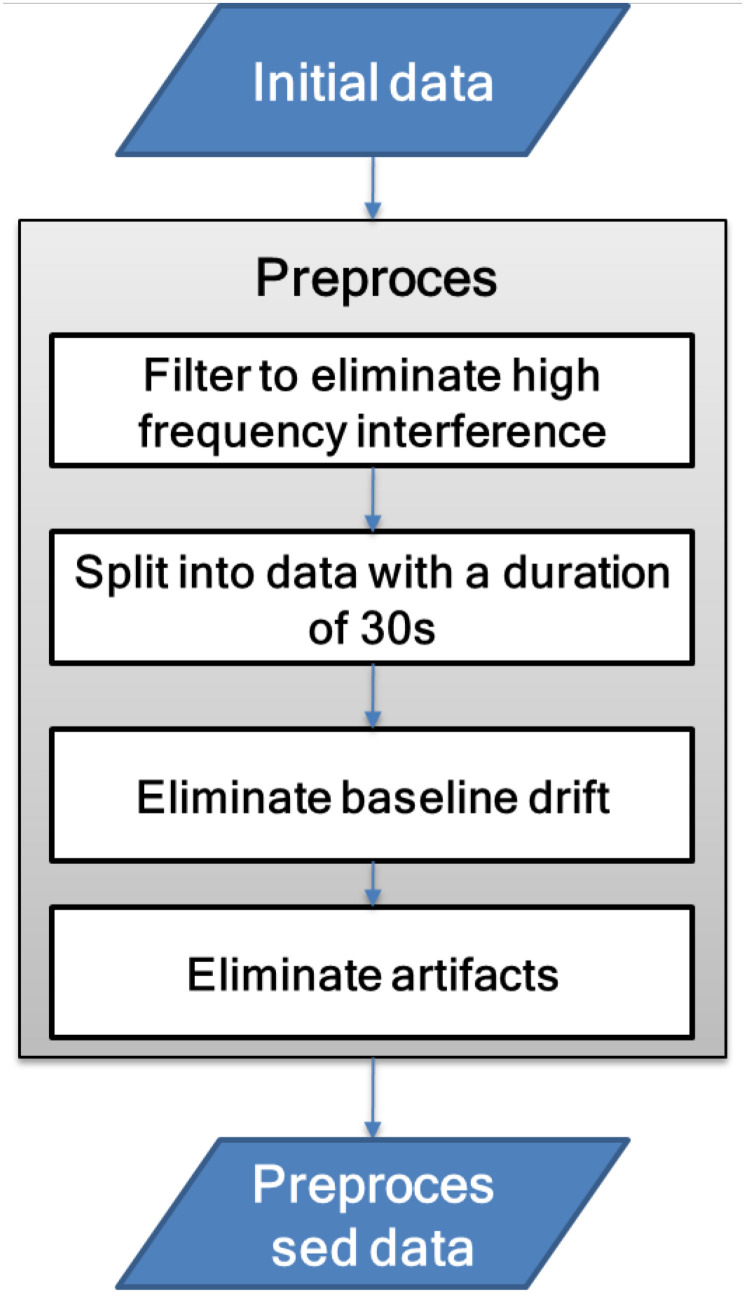
Signal preprocessing flowchart.

EEG is an electrophysiological signal with weak amplitude and is extremely susceptible to noise interference. Therefore, before signal analysis, it needs to be preprocessed to reduce high-frequency noise, baseline drift, and artifact interference. The signal preprocessing process is shown in Figure 3.

### Brain Wave Feature Extraction

This study uses discrete wavelet transform (DWT) for feature extraction. When extracting, the artificial sleep staging results obtained according to the AASM rules are used as the reference standards. When DWT analyzes the EEG signals, the main problem to be solved is the choice of decomposition layers and wavelet basis, where the decomposition layers are determined by the original signal frequency. The power of the EEG signal is mainly concentrated in the range of 0–30 Hz, so the decomposition frequency is set to 4 to extract all the characteristic frequency bands of the EEG signal. The signal is decomposed into D1–D4 components with detailed information and A4 components with low-frequency information.

**FIGURE 4:**
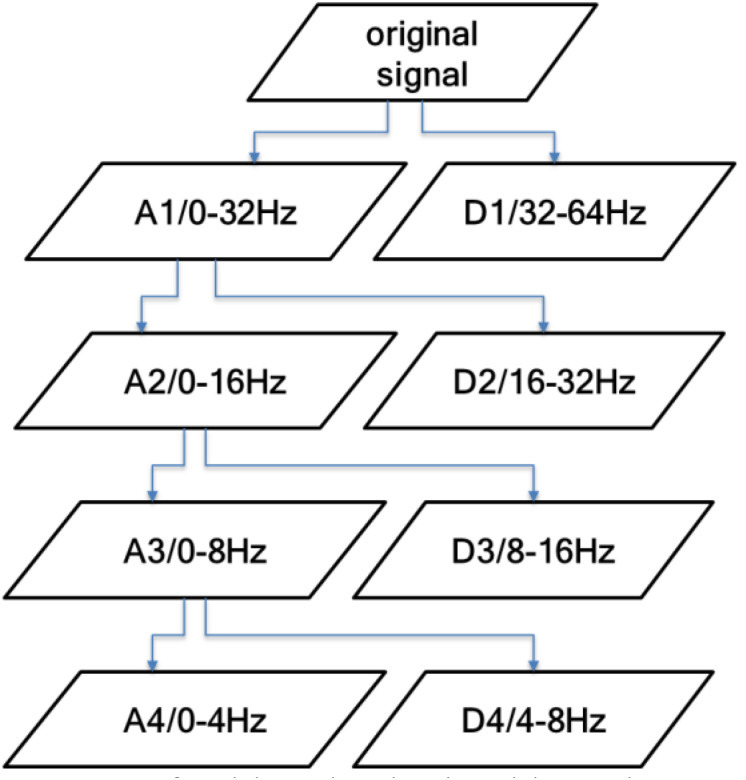
Frequency range of the sub-bands obtained by a four-layer discrete wavelet transform (DWT) decomposition.

Figure 4 shows the frequency range of the sub-band obtained by a four-layer DWT decomposition of the EEG signal. It can be seen from the figure that the A4 component contains the δ frequency band (0–4 Hz), the D4 component contains the θ frequency band (4–8 Hz), the D3 component contains the α frequency band (8–13 Hz) and part of the β frequency band, and the D2 component contains the β frequency band (13–30 Hz). The D1 component has frequency information higher than 30 Hz, which basically contains no information about the EEG signal. Therefore, in this study, the D2–D4 detailed component and the low-frequency component A4 are used.

In this paper, the db4 wavelet is used to decompose the EEG signal into four layers, and the mean and standard deviation of the absolute values of the D2–D4 and A4 components are counted. Because of the particularity of wavelet decomposition, first calculate the wavelet coefficients on the 25-s timescale and then use the sliding window to obtain the parameters of 80, 140, and 200 s by calculating the mean. In this paper, the four-layer wavelet decomposition of the db4 wavelet is used to process the EEG signals, and the wavelet coefficients of the four frequency bands shown in Table 3 are extracted.

**TABLE 3:** Correlation between wavelet components and the EEG signal frequency band.

Component	Frequency range (Hz)
A4	δ frequency band
D2	β frequency band
D3	α and part of β frequency band
D4	θ frequency band

### Sleep Brain Wave Classifier Training

The classifier used in this paper is TSVM. It uses relevant knowledge of the source domain to assist the target domain in establishing a classification model. Among them, a large number of labeled sample sets (*T*_s_) in the source domain are similar to the target domain test set (Test), and a small number of the labeled sample sets in the target domain (*T*_t_) are the same as the Test. By “transferring” *T*_s_’s knowledge, *w*_s_, to *T*_t_, a classification model was obtained, *f*:*X* → *Y*, so that *f* could correctly classify Test.

**FIGURE 5:**
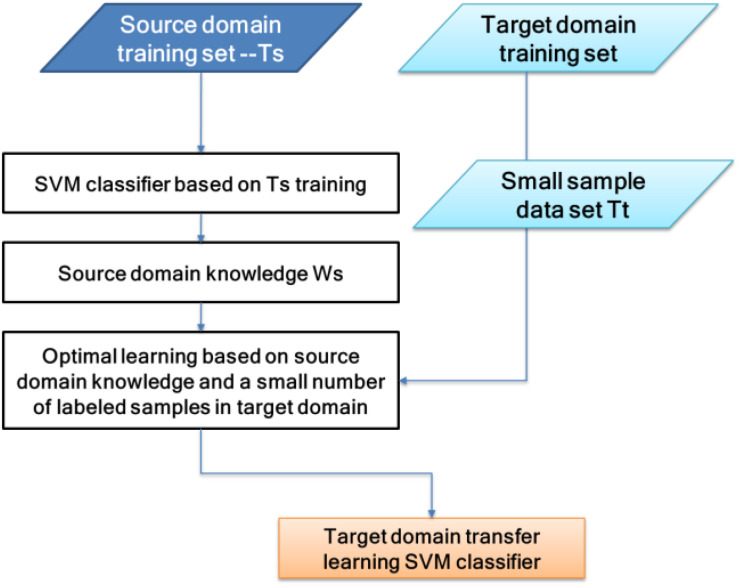
Transfer support vector machine (TSVM) principle diagram.

The SVM classifier consists of (*w*, *b*), the discriminant function is *f*(*x*) = *w*T_x_ +*b*, and the classification decision function is *L*(*x*) = sign(*f* (*x*)). The theoretical basis of the algorithm in this paper is that, if the two domains are related, the respective values of the two domain classifiers should be similar. By adding μ∥*w*_*t*_−*w*_*s*_∥^2^ to the SVM objective formula, transfer learning between the two domains can be achieved, where ∥*w*_*t*_−*w*_*s*_∥^2^ represents the degree of difference between the two-domain classifiers. The larger the value, the greater the difference between the classifiers. Parameter μ controls the penalty level. The principle of TSVM can be expressed in Figure 5.

There is a source domain SVM classifier (*w*_s_, *b*_s_). Use the source domain classifier knowledge, *w*_s_, to carry out transfer learning on the target domain. The optimization goal problem is as follows:

(1) $$\begin{array}{ccc} \min_{w_{t},b_{t}} & & \frac{1}{2}{∥w_{t}∥}^{2}+C_{t}\sum_{i=1}^{n}\xi_{i}^{t}+\mu {∥w_{t}-w_{s}∥}^{2}\\ s.t. & & y_{i}^{t}\left(\left(w_{t}\cdot x_{i}^{t}\right)+b_{t}\right)\geq 1-\xi_{i}^{t},i=1,2,\ldots ,n\\ & & \xi_{i}^{t}\geq 0,i=1,2,\ldots n. \end{array}$$

where β = (β_1_, β_2_,…, β*_*n*_*)^*T*^ and γ = (γ_1_, γ_2_,…, γ*_*n*_*)^*T*^ are the Lagrange multiplier column vectors. Find the partial derivatives of the original variables *w*_t_, $\xi_{i}^{t}$, and *b*_t_ and set to 0.

(2) $$\frac{\partial L}{\partial \xi_{i}^{t}}=C_{t}-\beta_{i}-\lambda_{i}=0\Rightarrow 0\leq \beta_{i}\leq C_{t}$$

(3) $$\frac{\partial L}{\partial w_{t}}=0\Rightarrow w_{t}=\frac{2\mu w_{s}+\sum_{i=1}^{n}\beta_{i}\left(y_{i}^{t}\cdot x_{i}^{t}\right)}{2\mu +1}$$

(4) $$\frac{\partial L}{\partial b_{t}}=0\Rightarrow \sum_{i=1}^{n}\beta_{i}y_{i}^{t}=0$$

Substituting Equations (3, 4) into the objective function (1), the dual form of the original problem is

(5) $$\begin{array}{c} \min_{\beta }\frac{1}{2\left(2\mu +1\right)}\sum_{i=1}^{n}\sum_{j=1}^{n}\beta_{i}\beta_{j}y_{i}^{t}y_{j}^{t}\left(x_{i}^{t}\cdot x_{j}^{t}\right)\\ +\sum_{i=1}^{n}\left(\frac{2\mu y_{i}^{t}\left(x_{i}^{t}\cdot w_{s}\right)}{2\mu +1}-1\right)\beta_{i}-\frac{\mu }{2\mu +1}{∥w_{s}∥}^{2}\\ s.t.0\leq \beta_{i}\leq C_{t},\sum_{i=1}^{n}\beta_{i}y_{i}^{t}=0,i=1,2,\ldots ,n. \end{array}$$

The specific algorithm of the transfer learning target domain classifier is as follows:

(1) Gain knowledge of the source domain *w*_s_, choose the appropriate penalty parameters *C*_t_, μ.
(2) Construct Equation (5) convex quadratic programming problem, obtain the solution β^∗^ = (β_1_^∗^,β_2_^∗^,…, β*_*n*_*^∗^)^*T*^, and obtain *w*_t_^∗^ by Equation (3).
(3) Select the β^∗^ component β_*j*_^∗^ in the open interval (0, *C*_t_) and calculate *b*_t_^∗^ = *y*_*j*_^*t*^ – (*w*_t_^∗^ ⋅ *x*_t_^*j*^).
(4) Construct a hyperplane (*w*_t_^∗^ ⋅ *x*_t_) + *b*_t_^∗^ = 0. From this, the decision function *f*(*x*^*e*^) = sign(*g*(*x*^*e*^)) is obtained, where *g*(*x*^*e*^) = (*w*_t_^∗^ ⋅ *xe*) + *b*^∗^.

## Experimental Verification and Results Analysis

### Experimental Data

The experimental data for this study come from the National Sleep Research Resource Library (NSRR). The resource library provides a large number of physiological signal data of clinical trials. The collected physiological signal data from The Sleep Heart Health Study (SHHS) implemented by the National Heart Lung & Blood Institute (NHLBI) of the United States are shared by the resource library. The samples of the polysomnography (PSG) experiment in the Sleep Heart Health Study (SHHS) dataset originated from 6, 441 individuals collected from 1995 to 1998. The subjects were all over 40 years old and in good health. In this paper, the first 60 sets of samples in the Sleep Heart Health Study (SHHS) dataset will be used as the experimental test data. We randomly selected 40 as the training set and the remaining 20 as the test set.

### Evaluation Index

The evaluation indicators used in this article are shown in Table 4.

**TABLE 4:** Evaluation indicators.

Index	Index calculation formula	Description
True positive rate (recall)	$TPR=\frac{TP}{TP+FN}$	TP: true positive TN: true negative FP: false positive FN: false negative
True negative rate (specificity)	$TNR=\frac{TN}{TN+FP}$	
Precision	$precision=\frac{TP}{TP+FP}$	

### Experimental Results and Analysis

In order to verify the effectiveness of the sleep staging method proposed in this paper, we used the DWT feature extraction method. The comparative classifiers used are SVM (Melgani and Bruzzone, 2004), Bayes classifier (BC) (Moraes et al., 2020), decision tree (DT) classifier (Friedl and Brodley, 1997), transfer component analysis (TCA) (Abid et al., 2016), and joint distribution adaptation (JDA) (Xie et al., 2018). The parameters of the TCA algorithm are set as: regularization parameter λ ∈ {0.01,…, 100} and dimension parameter dim ∈ {10,…, 100}. The parameters of the JDA algorithm are set as: regularization parameter λ ∈ {0.01,…, 100}, dimension parameter dim ∈ {10,…, 100}, and iteration parameter iter = {1,3,5}. The value range of the parameter μ in the TSVM algorithm is {0.001,0.005,0.1,0.3,0.5,0.7,0.9,0.99}. Tables 5–7 are comparisons of the evaluation indexes TPR, TNR, and precision of the test dataset under different classifiers. Only some use cases are given in each table.

**TABLE 5:** TPR indicators of the different classifiers on the test dataset (in percent).

Number model	SVM	BC	DT	TCA	JDA	TSVM
200070	87.71	81.20	78.73	91.32	91.56	90.57
200071	83.39	77.88	80.21	90.74	93.91	93.20
200072	82.81	78.70	77.75	86.51	86.67	85.70
200073	82.78	82.32	83.31	85.93	86.38	87.11
200077	83.22	58.98	81.23	89.39	89.51	88.87
200076	87.35	78.87	75.32	86.63	85.22	85.05
200077	83.57	83.38	80.27	90.12	87.09	86.18
200078	82.83	80.20	79.57	86.78	86.43	87.20
200078	83.21	72.32	83.53	84.35	87.58	86.78
200079	83.20	78.57	81.10	88.96	87.77	87.00
200080	82.87	80.55	83.37	89.23	88.80	87.27
200081	83.57	81.79	87.38	87.10	89.92	89.85
200082	87.78	75.37	75.77	86.54	89.53	90.33
200083	87.79	57.94	57.73	87.96	91.22	91.78
200084	83.52	81.18	78.75	88.92	92.81	90.80
200085	82.27	78.78	81.33	88.61	87.62	87.07
200086	83.72	81.10	82.10	87.43	86.32	88.17
200087	82.51	78.75	78.75	82.89	84.46	83.37
200088	83.23	81.52	78.80	86.37	86.59	85.78
200089	83.78	77.88	87.23	86.66	88.11	89.00
Mean	84.06	77.36	79.61	87.62	88.38	88.05

**TPR, true positive rate; SVM, support vector machine; BC, Bayes classifier; DT, decision tree; TCA, transfer component analysis; JDA, joint distribution adaptation; TSVM, transfer support vector machine.**

**TABLE 6:** TNR indicators of the different classifiers on the test dataset (in percent).

Number model	SVM	BC	DT	TCA	JDA	TSVM
200070	91.12	92.20	91.03	95.82	95.95	94.55
200071	93.39	89.99	91.21	94.91	95.03	96.22
200072	92.91	93.10	91.15	93.23	93.56	95.00
200073	90.29	92.32	92.30	93.68	95.11	94.18
200077	91.20	89.99	90.26	93.85	97.36	96.91
200076	92.35	92.91	94.32	96.35	96.94	97.05
200077	93.55	94.39	92.51	97.10	96.12	96.17
200078	95.43	94.27	93.51	98.51	98.79	96.20
200078	93.21	91.32	93.43	96.56	97.20	97.57
200079	94.20	89.51	90.00	96.02	97.46	96.00
200080	92.91	90.55	93.22	95.44	98.02	97.21
200081	93.52	92.17	91.47	93.87	95.33	96.95
200082	90.59	94.31	93.11	94.67	96.63	96.33
200083	91.37	92.94	91.13	94.90	95.35	95.19
200084	93.52	91.16	93.15	95.34	95.76	96.90
200085	92.20	94.19	94.03	95.89	96.54	97.01
200086	93.12	91.10	92.30	94.17	96.10	95.11
200087	92.51	89.15	92.15	94.63	97.27	96.31
200088	95.23	91.50	93.90	93.43	96.21	95.19
200089	93.19	90.99	91.53	96.14	95.92	96.06
Mean	92.79	91.90	92.29	95.23	96.33	96.11

**TNR, true negative rate; SVM, support vector machine; BC, Bayes classifier; DT, decision tree; TCA, transfer component analysis; JDA, joint distribution adaptation; TSVM, transfer support vector machine.**

**TABLE 7:** Precision indicators of different classifiers on the test dataset (in percent).

Number model	SVM	BC	DT	TCA	JDA	TSVM
200070	72.45	71.20	67.43	74.60	73.47	73.54
200071	73.10	69.78	70.21	73.75	74.07	74.20
200072	72.71	68.90	69.65	72.08	72.84	73.90
200073	72.67	72.32	73.31	72.43	75.85	74.11
200077	73.22	58.67	71.23	73.66	74.11	74.89
200076	74.35	68.89	65.32	75.28	75.23	75.05
200077	73.56	73.37	70.26	73.60	74.81	75.17
200078	72.73	70.20	69.56	73.98	75.92	74.20
200078	73.21	62.32	73.53	75.73	72.35	74.98
200079	73.20	68.59	71.10	73.68	74.22	75.00
200080	72.76	80.56	73.34	73.52	75.02	74.29
200081	73.54	71.44	74.38	73.80	74.33	74.85
200082	76.48	65.34	65.94	75.74	76.94	78.33
200083	76.64	59.69	59.43	75.47	78.90	77.97
200084	73.52	71.17	68.65	76.38	75.87	75.80
200085	72.19	68.98	71.33	74.11	75.68	74.09
200086	73.92	81.10	72.10	75.92	75.05	76.16
200087	72.41	68.65	67.45	74.22	74.88	73.39
200088	73.26	71.52	68.80	74.26	76.43	75.98
200089	73.48	69.78	74.23	75.89	77.31	76.00
Mean	73.47	69.62	69.86	74.41	75.16	75.10

**SVM, support vector machine; BC, Bayes classifier; DT, decision tree; TCA, transfer component analysis; JDA, joint distribution adaptation; TSVM, transfer support vector machine.**

An algorithm with excellent performance should have higher TPR, TNR, and precision. As can be seen from the data in Tables 5–7, on the indicator TPR, the TSVM algorithm improves the traditional SVM, BC, and DT by 4.7, 13.8, and 11.0%, respectively. On the indicator TNR, it increased by 3.6, 4.6, and 4.1%, respectively. On the indicator precision, it increased by 2.2, 7.9, and 5.2%, respectively. The performance of the SVM algorithm is more stable and better than those of the BC and DT algorithms, which is why we chose SVM as the basic algorithm. The performance of the TSVM algorithm used in this article is ahead of the comparison algorithm in all three evaluation indicators. This is because the introduction of the transfer mechanism can effectively utilize useful information from the source domain data and improve the classification performance.

Comparing the three migration learning algorithms TCA, JDA, and TSVM, the performance gap of each algorithm is not big. Among them, the performance of the JDA algorithm is the best, the TSVM used in this article is the second, and TCA is the worst. The reasons for choosing TSVM in this article are as follows: firstly, the TSVM algorithm is more widely used, and the mathematical principles and implementation process are relatively simple. Secondly, compared with other migration algorithms, TSVM has little performance gap, and the recognition results based on TSVM can fully meet the needs of reality. Thirdly, TSVM needs to optimize and set a few parameters, but JDA, which has the best classification effect, needs to optimize and set many parameters. If the parameters are selected differently, the final operation effect of the algorithm will be very different. Based on the above reasons, it is feasible to choose TSVM as the final classifier in this paper.

## Conclusion

In order to check the quality of sleep, this paper mainly carried out research work on sleep staging. The innovation of this research lies in the introduction of a transfer learning classifier, which can effectively improve the classification performance of the data. The transfer learning classifier introduces the transfer learning mechanism based on the traditional SVM classifier. The introduction of the transfer learning mechanism can effectively use the knowledge of the source domain to guide the classification task of the target domain. In sleep staging research work, the EEG signal is first preprocessed, then DWT is used for feature extraction, and, finally, the TSVM with transfer learning mechanism is used to classify the feature data. The experimental results on the public dataset show that the method in this paper has greatly improved the performance of classification and can achieve the detection of sleep quality to a certain extent. However, this article only uses EEG signals for research, which has limitations. The research on sleep quality based on multimodal physiological signals can be expanded in the future.

## Data Availability Statement

The original contributions presented in the study are included in the article/supplementary material, further inquiries can be directed to the corresponding author/s.

## Author Contributions

WW independently conceived and designed the framework of the manuscript. From the determination of the research problem, the selection and implementation of the solution are all done independently by WW. During the implementation of the solution, the main tasks include model training, experimental data evaluation and analysis, and manuscript preparation. WW was independently responsible for the entire process from conception to the completion of the manuscript.

## Conflict of Interest

The author declares that the research was conducted in the absence of any commercial or financial relationships that could be construed as a potential conflict of interest.

## References

[B1] Abid F. Hassan A. Abid A. Niazi I. K. Jochumsen M. (2016). “Transfer learning for electroencephalogram signals,” in *Proceedings of the 9th International Conference on Computer and Electrical Engineering (ICCEE)*, Barcelona.

[B2] Ahmed M. N. Yamany S. M. Mohamed N. Farag A. A. Moriarty T. (2002). Amodified fuzzy c-means algorithm for bias field estimation and segmentation of MRI data. *IEEE Trans. Med. Imaging* 21 193–199. 10.1109/42.996338 11989844

[B3] Albayrak M. Koklukaya E. (2009). The detection of an epileptiform activity on EEG signals by using data mining process. *E J. New World Sci. Acad.* 4 1–12.

[B4] Alessandro M. D. Vachtsevanos G. Hinson A. Esteller R. (2001). “A genetic approach to selecting the optimal feature for epileptic seizure prediction,” in *Proceedings of the 23rd Annual International Conference of the IEEE on Engineering in Medicine and Biology Society*, Istanbul.

[B5] Baja V. Pachori R. B. (2013). Analysis and classification of sleep stages based on difference visibility graphs from a single-channel EEG signal. *Comput. Methods Program. Biomed.* 112 320–328.

[B6] Bezdek J. C. (1980). A convergence theorem for the fuzzy ISODATA clustering algorithm. *IEEE Trans. Pattern Analys. Mach. Intellig.* 1 1–8. 10.1109/TPAMI.1980.4766964 22499617

[B7] Bezdek J. C. (1981). *Pattern Recognition with Fuzzy Objective Function Algorithms.* New York, NY: Plenum Press.

[B8] Bruzzo A. A. Gesierich B. Santi M. Tassinari C. A. (2008). Permutation entropy to detect vigilance changes and preictal states from scalp EEG in epileptic patients-A preliminary study. *Neurol. Sci.* 29 3–9. 18379733 10.1007/s10072-008-0851-3

[B9] Cai T. T. Ma J. Zhang L. (2019). Chime: clustering of high-dimensional Gaussian mixtures with EM algorithm and its optimality. *Ann. Statist.* 47 1234–1267. 10.1214/18-AOS1711

[B10] Chen S. Zhang D. (2004). Robust image segmentation using FCM with spatialconstraints based on new kernel-induced distance measure. *IEEE Trans. Syst. Man Cybernet. Part B Cybernet.* 34 1907–1916. 10.1109/TSMCB.2004.831165 15462455

[B11] Chen X. Xu X. Huang J. Z. Ye Y. (2013). TW-k-means: automated two-level variable weighting clustering algorithm for multiview data. *IEEE Trans. Knowl. Data Eng.* 25 932–944. 10.1109/TKDE.2011.262

[B12] Chrobak M. Dürr C. Aleksander F. Nilsson B. J. (2020). Online clique clustering. *Algorithmica* 82 938–965. 10.1007/s00453-019-00625-1

[B13] Chuang K. S. Tzeng H. L. Chen S. Wu J. Chen T. J. (2006). Fuzzy c-means clustering with spatial information for image segmentation. *Comput. Med. Imaging Graph. Off. J. Comput. Med. Imaging Soc.* 30 9–15. 10.1016/j.compmedimag.2005.10.001 16361080

[B14] Cleuziou G. Exbrayat M. Martin L. Sublemontier J. H. (2009). “Co FKM: a centralized method for multiple-view clustering,” in *Proceedings of the 9th International Conference on Data Mining*, Miami FL, 10.1109/ICDM.2009.138

[B15] Devi B. R. Setty S. P. (2018). Hybrid clustering algorithm ‘KCu’ for combining the features of K-means and CURE Algorithm for efficient outliers handling. *Adv. Model. Analys. B* 61 76–79. 10.18280/ama_b.610204

[B16] Doroshenkov L. G. Konyshev V. A. Selishchev S. V. (2007). Classification of human sleep stages based on EEG processing using hidden markov models. *Biomed. Eng.* 41 25–28. 17419342

[B17] Elazab A. Wang C. Jia F. Wu J. Li G. Hu Q. (2015). Segmentation of brain tissues from magnetic resonance images using adaptively regularized kernel-based fuzzy c-means clustering. *Comput. Math. Methods Med.* 2015 1–12. 10.1155/2015/485495 26793269 PMC4697674

[B18] Fathima T. Bedeeuzzaman M. Farooq O. (2010). Wavelet based features for epileptic seizure detection. *MES J. Technol. Manag.* 2 108–112.

[B19] Fraiwan L. Lweesy K. Khasawneh N. Fraiwan M. Wenz H. Dickhaus H. (2010). Classification of sleep stages using multi-wavelet time frequency entropy and LDA. *Methods Inform. Med.* 49:230. 10.3414/ME09-01-0054 20091018

[B20] Fraiwan L. Lweesy K. Khasawneh N. Wenz H. Dickhaus H. (2012). Automated sleep stage identification system based on time-frequency analysis of a single EEG channel and random forest classifier. *Comput. Methods Prog. Biomed.* 108 10–19. 10.1016/j.cmpb.2011.11.005 22178068

[B21] Friedl M. A. Brodley C. E. (1997). Decision tree classification of land cover from remotely sensed data. *Remote Sens. Environ.* 61 399–409.

[B22] Gandhi T. K. Chakraborty P. Roy G. G. Panigrahi B. K. (2012). Discrete harmony search based expert model for epileptic seizure detection in electroencephalography. *Expert Syst. Appl.* 39 4055–4062.

[B23] Geng S. Zhou W. Yuan Q. Cai D. Zeng Y. (2011). EEG non-linear feature extraction using correlation dimension and Hurst exponent. *Neurol. Res.* 33 908–912. 22080990 10.1179/1743132811Y.0000000041

[B24] Gong M. Liang Y. Shi J. Ma W. Ma J. (2013). Fuzzy c-means clustering with local information and kernel metric for image segmentation. *IEEE Trans. Image Process.* 22 573–584. 10.1109/TIP.2012.2219547 23008257

[B25] Gu J. Cheng T. Hua L. Wang J. Zhao J. Cao Y. (2019). Overview of image segmentation and registration for spine biological modeling. *J. Syst. Simulat.* 31 167–173. 10.16182/j.issn1004731x.joss.18-0806

[B26] Gu Q. Zhou J. (2009). “Learning the shared subspace for multi-task clustering and transductive transfer classification,” in *Proceedings of the IEEE International Conference on Data Mining*, Miami Beach, FL, 10.1109/ICDM.2009.32

[B27] Hall L. O. Bensaid A. M. Clarke L. P. (1992). A comparison of neural network and fuzzy clustering techniques in segmenting magnetic resonance images of the brain. *IEEE Trans. Neural Netw.* 3 672–681. 10.1109/72.159057 18276467

[B28] Hall L. O. Goldgof D. B. (2011). Convergence of the single-pass and online fuzzy C-means algorithms. *IEEE Trans. Fuzzy Syst.* 19 792–794. 10.1109/TFUZZ.2011.2143418

[B29] Hassan A. R. Bhuiyan M. I. H. (2016). Automatic sleep scoring using statistical features in the EMD domain and ensemble methods. *Biocybernet. Biomed. Eng.* 36 248–255.

[B30] Hsu Y. L. Yang Y. T. (2013). Automatic sleep stage recurrent neural classifier using energy features of EEG signals. *Neurocomputing* 104 105–114.

[B31] Huang C. S. Lin C. L. Ko L. W. Liu S. Y. Su T. P. Lin C. T. (2014). Knowledge-based identification of sleep stages based on two forehead electroencephalogram channels. *Front. Neurosci.* 8:263. 10.3389/fnins.2014.00263 25237291 PMC4154530

[B32] Ji Z. X. Sun Q. S. Xia D. S. (2011). A modified possibilisticfuzzy c-means clustering algorithm for bias field estimation and segmentation of brain MR image. *Comput. Med. Imag. Graph.* 35 383–397. 10.1016/j.compmedimag.2010.12.001 21256710

[B33] Jiang C. F. Chang C. C. Huang S. H. (2012). Regions of interest extraction fromspect images for neural degeneration assessment using multimodality image fusion. *Multidimens. Syst. Signal. Process.* 23 437–449. 10.1007/s11045-011-0162-3

[B34] Jiang Y. Bi A. Xia K. Xue J. Qian P. (2020). Exemplar-based data stream clustering toward internet of things. *J. Supercomput.* 76 2929–2957. 10.1007/s11227-019-03080-5

[B35] Jiang Y. Chung F. L. Wang S. Deng Z. Wang J. Qian P. (2014). Collaborative fuzzy clustering from multiple weighted views. *IEEE Trans. Cybernet.* 45 688–701. 10.1109/TCYB.2014.2334595 25069132

[B36] Jiang Y. Deng Z. Chung F. L. Wang G. Qian P. Choi K. S. (2017a). Recognition of epileptic EEG signals using a novel multiview TSK fuzzy system. *IEEE Trans. Fuzzy Syst.* 25 3–20. 10.1109/TNSRE.2017.2748388 28880184

[B37] Jiang Y. Wu D. Deng Z. Qian P. Wang J. Wang G. (2017b). Seizure classification from EEG Signals using transfer learning, semi-supervised learning and TSK fuzzy system. *IEEE Trans. Neural Syst. Rehabil. Eng.* 25 2270–2284. 28880184 10.1109/TNSRE.2017.2748388

[B38] Jiang Y. Zhao K. Xia K. Xue J. Zhou L. Ding Y. (2019). A Novel distributed multitask fuzzy clustering algorithm for automatic MR brain image segmentation. *J. Med. Syst.* 43:118. 10.1007/s10916-019-1245-1 30911929

[B39] Jing L. P. Ng M. K. Huang J. Z. (2007). An entropy weighting k-means algorithm for subspace clustering of high-dimensional sparse data. *IEEE Trans. Knowl. Data Eng.* 19 1026–1041. 10.1109/TKDE.2007.1048

[B40] Kannathal N. Choo M. Acharya U. Sadasivan P. (2005). Entropies for detectio of epilepsy in EEG. *Comput. Methods Prog. Biomed.* 80 187–194. 10.1016/j.cmpb.2005.06.012 16219385

[B41] Kayikcioglu T. Maleki M. Eroglu K. (2015). Fast and accurated PLS-based classification of EEG sleep using single channel data. *Expert Syst. Appl.* 42 7825–7830.

[B42] Koch H. Christensen J. A. (2014). Automatic sleep classification using a data-driven topic model reveals latent sleep states. *J. Neurosci. Methods* 235 130–137. 25016288 10.1016/j.jneumeth.2014.07.002

[B43] Krinidis S. Chatzis V. (2010). A robust fuzzy local information C-means clustering algorithm. *IEEE Trans. Image Process.* 19 1328–1337. 10.1109/TIP.2010.2040763 20089475

[B44] Kumar S. N. Lenin F. A. Sebastin V. P. (2020). An overview of segmentation algorithms for the analysis of anomalies on medical images. *J. Intellig. Syst.* 29 612–625. 10.1515/jisys-2017-0629

[B45] Lajnef T. Chaibi S. Ruby P. Aguera P. E. Eichenlaub J. B. Samet M. (2015). Learning machines and sleeping brains: automatic sleep stage classification using decision-tree multi-class support vector machines. *J. Neurosci. Methods* 250 94–105. 25629798 10.1016/j.jneumeth.2015.01.022

[B46] Lee K. Moon C. Nam Y. (2018). Diagnosing vocal disorders using cobweb clustering of the jitter, shimmer, and harmonics-to-noise ratio. *KSII Trans. Internet Inform. Syst.* 12 5541–5554. 10.3837/tiis.2018.11.020

[B47] Li C. Gore J. C. Davatzikos C. (2014). Multiplicative intrinsic component optimization(MICO). for MRI bias field estimation and tissue segmentation. *Magnet. Resonan. Imaging* 32 413–439. 10.1016/j.mri.2014.03.010 24928302 PMC4401088

[B48] Liu L. Kuang L. Ji Y. (2020). Multimodal MRI brain tumor image segmentation using sparse subspace clustering algorithm. *Computat. Math. Methods Med.* 2020:8620403. 10.1155/2020/8620403 32714431 PMC7355351

[B49] Long X. Foussier J. Fonseca P. Haakma R. Aarts R. M. (2014). Analyzing resporatory effort amplitude for automated sleep stage classification. *Biomedical Signal Process. Control* 14 197–205.

[B50] Melgani F. Bruzzone I. (2004). Classification of hyperspectral remote sensing images with support vector machines. *IEEE Transgeosci. Remote Sens.* 42 1778–1790.

[B51] Mohseni H. R. Maghsoudi A. Shamsollah I. M. B. (2006). “Seizure detection in EEG signals: a comparison of different approaches,” in *Proceedings of the 28th Annual International Conference of the IEEE on Engineering in Medicine and Biology Society*, New York, NY. 10.1109/IEMBS.2006.260931 17959496

[B52] Moraes R. M. Soare E. A. M. G. Machado L. S. Machado L. S. Kahraman C. (2020). A double weighted fuzzy gamma naive bayes classifier. *J. Intellig. Fuzzy Syst.* 38 577–588.

[B53] Okita Y. Miyata A. H. Motomura B. N. Takamoto S. (2015). A study of brain protection during total arch replacement comparing antegrade cerebral perfusion versus hypothermic circulatory arrest, with or without retrograde cerebral perfusion: analysis based on the Japan adult cardiovascular surgery database. *J. Thorac. Cardiovasc. Surg.* 149 65–73. 10.1016/j.jtcvs.2014.08.070 25439767

[B54] Pedrycz W. (2002). Collaborative fuzzy clustering. *Pattern Recogn. Lett.* 23 1675–1686. 10.1016/S0167-865502.00130-7

[B55] Qian P. Chen Y. Kuo J. W. Zhang Y. D. Jiang Y. Zhao K. (2020). mDixon-based synthetic CT generation for PET attenuation correction on abdomen and pelvis jointly using transfer fuzzy clustering and active learning-based classification. *IEEE Trans. Med. Imaging* 39 819–832. 31425065 10.1109/TMI.2019.2935916 PMC7284852

[B56] Qian P. Jiang Y. Deng Z. Hu L. Sun S. Wang S. (2015). Cluster prototypes and fuzzy memberships jointly leveraged cross-domain maximum entropy clustering. *IEEE Trans. Cybernet.* 46 181–193. 10.1109/TCYB.2015.2399351 26684257 PMC4882931

[B57] Qian P. Jiang Y. Wang S. Su K. H. Wang J. Hu L. (2016a). Affinity and penalty jointly constrained spectral clustering with all-compatibility, flexibility, and robustness. *IEEE Trans. Neural Netw. Learn. Syst.* 28 1123–1138. 10.1109/TNNLS.2015.2511179 26915134 PMC4990515

[B58] Qian P. Sun S. Jiang Y. Su K. H. Ni T. Wang S. (2016b). Cross-domain, soft-partition clustering with diversity measure and knowledge reference. *Pattern Recogn.* 50 155–177. 10.1016/j.patcog.2015.08.009 PMC4892128 27275022

[B59] Qian P. Xi C. Xu M. Jiang Y. Su K. H. Wang S. (2018a). SSC-EKE: semi-supervised classification with extensive knowledge exploitation. *Inform. Sci.* 422 51–76. 10.1016/j.ins.2017.08.093 PMC5881956 29628529

[B60] Qian P. Zhou J. Jiang Y. Liang F. Muzic R. F. (2018b). Multi-view maximum entropy clustering by jointly leveraging inter-view collaborations and intra-view-weighted attributes. *IEEE Access.* 6 28594–28610. 10.1109/ACCESS.2018.2825352 31289704 PMC6615759

[B61] Qian P. Zhao K. Jiang Y. Su K. H. Deng Z. Wang S. (2017). Knowledge-leveraged transfer fuzzy C -Means for texture image segmentation with self-adaptive cluster prototype matching. *Knowl. Based Syst.* 130 33–50. 30050232 10.1016/j.knosys.2017.05.018 PMC6056248

[B62] Rosati P. Lupascu C. A. Tegolo D. (2018). Analysis of low-correlated spatial gene expression patterns: a clustering approach in the mouse brain data hosted in the Allen Brain Atlas. *IET Comput. Vis.* 12 996–1006. 10.1049/iet-cvi.2018.5217

[B63] Sen B. Peker M. (2013). Novel approaches for automated epileptic diagnosis using FCBF feature selection and classification algorithms. *Turk. J. Electric. Eng. Comput. Sci.* 21 2092–2109.

[B64] Siegel J. M. (2005). Clues to the functions of mammalian sleep. *Nature* 437 1264–1271. 16251951 10.1038/nature04285 PMC8760626

[B65] Singh M. Vishal V. Verma A. Sharma N. (2020). Segmentation of MRI data using multi-objective antlion based improved fuzzy c-means. *Biocybernet. Biomed. Eng.* 40 1250–1266. 10.1016/j.bbe.2020.07.001

[B66] Srinivasan V. Eswaran C. Sriraam N. (2005). Artificial neural network based epileptic detection using time domain and frequency domain features. *J. Med. Syst.* 29 647–660. 16235818 10.1007/s10916-005-6133-1

[B67] Subasi A. (2007). EEG signal classification using wavelet feature extraction and a mixture of expert model. *Expert Syst. Appl.* 32 1084–1093.

[B68] Sunjana Azizah Z. (2020). Outlier detection of transaction data using DBSCAN Algorithm. *Intern. J. Psychosoc. Rehabil.* 24 3232–3340. 10.37200/IJPR/V24I2/PR200632

[B69] Thanh M. N. Wu Q. M. J. (2013). A fuzzy logic model based Markov random field for medical image segmentation. *Evolv. Syst.* 4 171–181. 10.1007/s12530-012-9066-1

[B70] Wang J. Schreiber D. K. Bailey N. Hosemann P. Toloczko M. B. (2019). The application of the OPTICS algorithm to cluster analysis in atom probe tomography data. *Microsc. Microanalys.* 25 338–348. 10.1017/S1431927618015386 30846021

[B71] Wang X. Y. Zhang D. D. Wei N. (2015). Fractal image coding algorithm using particle swarm optimisation and hybrid quadtree partition scheme. *IET Image Process.* 9 153–161. 10.1049/iet-ipr.2014.0001

[B72] Weijer J. Gevers T. (2006). Boosting color saliency in image feature. *IEEE Trans. Pattern Analys. Mach. Intellig.* 28 150–156. 10.1109/TPAMI.2006.3 16402628

[B73] Xia K. J. Zhong X. Zhang L. Wang J. (2019). Optimization of diagnosis and treatment of chronic diseases based on association analysis under the background of regional integration. *J. Med. Syst.* 43:46. 10.1007/s10916-019-1169-9 30661117

[B74] Xie L. Deng Z. Xu P. Choi K. S. Wang S. (2018). Generalized Hidden-mapping transductive transfer learning for recognition of epileptic electroencephalogram signals. *IEEE Trans. Cybernet.* 49 2200–2214. 10.1109/TCYB.2018.2821764 29993945

[B75] Yin S. Li H. Liu D. Karim S. (2020). Active contour modal based on density-oriented BIRCH clustering method for medical image segmentation. *Multimed. Tools Appl.* 79 31049–31068. 10.1016/j.ijleo.2018.01.004

[B76] Yu S. Tranchevent L. Liu X. Glanzel W. Suykens J. A. K. De Moor B. (2012). Optimized data fusion for kernel k-means clustering. *IEEE Trans. Pattern Analys. Mach Intellig.* 34 1031–1039. 10.1109/TPAMI.2011.255 22442124

[B77] Yuen C. T. San W. S. Rizoni M. Seong T. C. (2009). Classification of human emotions from EEG signals using statistical features and neural network. *Intern. J. Integrat. Eng.* 1 71–79.

[B78] Zhang C. Churazov E. Zhuravleva I. (2020). Pairs of giant shock waves (N-waves). in merging galaxy clusters. *Mon. Notic. R. Astron. Soc.* 501 1038–1045. 10.1093/mnras/staa3718

[B79] Zheng Y. Byeungwoo J. Xu D. Wu Q. M. J. Hu Z. (2015). Image segmentation by generalized hierarchical fuzzy C- means algorithm. *J. Intellig. Fuzzy Syst.* 28 4024–4028. 10.3233/IFS-141378

[B80] Zhu L. Chung F. L. Wang S. T. (2009). Generalized fuzzy k-means clustering algorithm with improved fuzzy partitions. *IEEE Trans. Syst. Man Cybernet.* 39 578–591. 10.1109/TSMCB.2008.2004818 19174354

